# Efficacy and safety of anti-PD-1/PD-L1 therapy in the treatment of advanced colorectal cancer: a meta-analysis

**DOI:** 10.1186/s12876-022-02511-7

**Published:** 2022-10-10

**Authors:** Yuegang Li, Yuwei Du, Chi Xue, Pei Wu, Nan Du, Guolian Zhu, Huimian Xu, Zhi Zhu

**Affiliations:** 1 Department of Surgical Oncology, The First Affiliated Hospital of China Medical University, Shenyang, 110001 China; 2 Department of Surgical Oncology, The Fifth People’s Hospital of Shenyang City, Shenyang, China

**Keywords:** PD-1, PD-L1, Immunotherapy, Colorectal cancer, Meta-analysis

## Abstract

**Background:**

Immune checkpoint inhibitors have shown promise in microsatellite instability-high/mismatch repair deficient (MSI-H/dMMR) advanced colorectal cancer (CRC) immunotherapy, and many clinical trials have been conducted.

**Objective:**

To evaluate the efficacy and safety of PD-1/PD-L1 inhibitors in advanced CRC.

**Method:**

PubMed, Web of Science, Embase, and The Cochrane Library were searched for relevant studies up to September 2021. A retrospective cross-sectional data analysis was performed and Stata 16 software was used for analyses.

**Results:**

Sixteen studies including 1503 patients were analyzed. The objective response rate (ORR) of anti-PD-1/PD-L1 was 23% (95% CI 0.14, 0.31); the overall 1-year survival rate (OSR) was 57% (95% CI 0.42, 0.73). The ORR of MSI-H/dMMR advanced CRC was 37% (95% CI 0.25, 0.48) and that of microsatellite stable/mismatch repair proficient (MSS/pMMR) disease was 11% (95% CI 0.06, 0.16). The ORR was 42% in the BRAF mutant subgroup and 19% in the RAS mutant group. The ORR was 14% in the PD-L1 ( +) subgroup and 32% in the PD-L1(-) subgroup. The rate of adverse effects was 85% (95% CI 0.80, 0.91).

**Conclusion:**

Anti-PD-1/PD-L1 therapy in MSI-H/dMMR advanced CRC was associated with improved survival. Anti PD-1/PD-L1 combined with antiangiogenic drugs, targeted agents, or chemotherapy might be effective in MSS mCRC. Immunotherapy was effective for the BRAF mutant and KRAS/NRAS(RAS) mutant CRC. Low expression of PD-L1 was a potential predictive marker for positive response and outcome. The high incidence of adverse events at 85% was worthy of further investigation. Further analysis with a higher number of high-quality studies is needed to verify the conclusions.

**Supplementary Information:**

The online version contains supplementary material available at 10.1186/s12876-022-02511-7.

## Background

Colorectal cancer (CRC) is the third most common cancer in the world and ranks second in mortality. Every year more than 800,000 people worldwide die from CRC [1]. The 5-year survival rate for patients with early CRC can reach 90% compared with 10% for metastatic CRC (mCRC) [2]. The standard chemotherapy for mCRC is fluoropyrimidine, oxaliplatin, and irinotecan-based combination regimens with anti-EGFR and anti-VEGF treatment [3–6]. This strategy has achieved positive short-term results, although the long-term efficacy is poor. Several recent studies show that the PD-1/PD-L1 pathway affects the balance between tumor immune escape and immune surveillance [7, 8]. Antibodies that block the PD-1/PD-L1 pathway have been approved for multiple solid tumors, including melanoma [9], lung cancer [10], head and neck cancer [11], urothelial cancer [12], Merkel cell carcinoma [13], and microsatellite instability-high/mismatch repair deficient (MSI-H/dMMR) mCRC. ASCO released the results of the KEYNOTE-177 study in 2020, which indicated that patients randomly assigned to receive pembrolizumab had significantly better overall survival (OS) and safety than patients receiving continued standard chemotherapy [14]. Although immunotherapy has achieved a certain efficacy in patients with MSI-H/dMMR mCRC, this population of patients accounts for 5% of mCRC patients, whereas 95% of mCRC patients present with microsatellite stable (MSS) disease. The KEYNOTE016 study showed that single immunotherapy was not effective for MSS mCRC patients [15]. Combination strategies with immunotherapy and the identification of predictive biomarkers for immunotherapy in MSS mCRC have become the focus of intense research efforts. However, most global studies on immunotherapy-related treatments for mCRC patients are single-arm and small clinical trials. Here, we performed a search of the relevant literature and conducted a meta-analysis of the efficacy and safety of anti-PD-1/PD-L1 therapy.

### Search strategy

We searched for eligible trials analyzing the use of anti-PD-1/PD-L1 therapy in the treatment of CRC published in PubMed, Web of Science, Embase, and The Cochrane Library from the date of their inception to Sep 1, 2021. The keywords used for the search were “colorectal neoplasms”, “colorectal cancer”, “colorectal tumors”, “colorectal carcinoma”, “immune checkpoint inhibitor”, “PD-1 Inhibitors”, “programmed cell death protein 1 inhibitor”, “PD-L1 Inhibitors”, and “Programmed Death-Ligand 1 Inhibitors”. We also searched the reference lists of retrieved articles to identify additional relevant publications.

### Inclusion and exclusion criteria

We included all articles focusing on analyzing the use of anti-PD-1/PD-L1 therapy in the treatment of CRC. The inclusion criteria were as follows: studies investigating the efficacy of anti-PD-1/PD-L1 agents with or without anti-CTLA-4 in the treatment of advanced CRC; only original research published in English was considered. The exclusion criteria were as follows: studies published as reviews, letters, case reports, animal studies, and meeting abstracts; studies with incomplete or inaccurate data for analysis.

### Data extraction

Two reviewers carried out the screening and extraction processes independently. Disagreements were resolved by discussion or by involving a third author. First, studies were screened by titles and abstracts. Then, the full text was read to determine whether it could be included. The data extracted included the following: (1) basic information of the study, including first author, publication year, country, study design, study interval, study objective, and sample size; (2) baseline characteristics of the research subjects, including the number of patients, age, the primary location and metastatic site of the tumor, microsatellite status, genotype, and PD-L1 status; and (3) outcome measures data. Results were checked by a third author.

### Quality evaluation

The quality of the studies was assessed in accordance with the ROBINS-I tool for observational studies and ROBINS-2 tool for randomized trials. Risk of bias for each item was graded as “low risk was defined as comparable to a well performed randomized trial with regard to this domain,” “moderate risk was defined as sound for a non-randomized study with regard to this domain, but not comparable to a well-performed randomized trial,” “serious risk was defined as presence of some important problems,” and “critical risk was defined as too problematic to provide any useful evidence on the effects of intervention.”

### Statistical analysis

The objective response rate (ORR), disease control rate (DCR), progression-free survival rate (PFSR), and the overall survival rate (OSR) with their 95% confidence intervals (CIs) were evaluated for the studies included in the meta-analysis. Heterogeneity in the outcomes was assessed using the χ^2^ and I^2^ tests. I^2^ > 50% and a *P*-value < 0.1 indicated significant heterogeneity, and the random-effects model was used. Otherwise, the fixed-effects model was used. Additional subgroup analysis was performed, and the results are described in detail. Funnel plots were generated to assess publication bias. All statistical analyses were performed using Stata16 software.

## Results

### Characteristics of studies

**Fig. 1:**
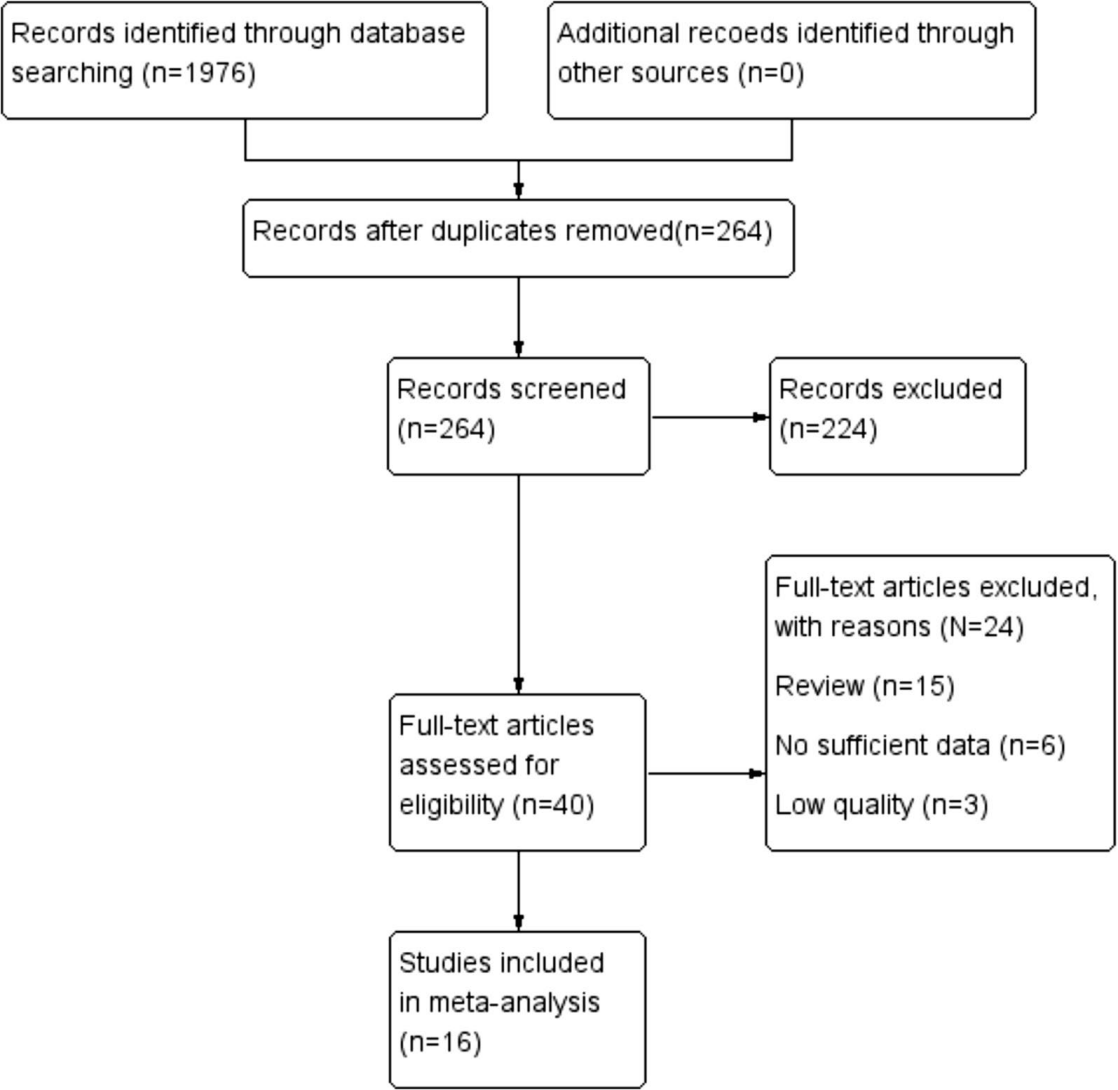
Flow diagram of the literature search in this meta-analysis

**Fig. 2:**
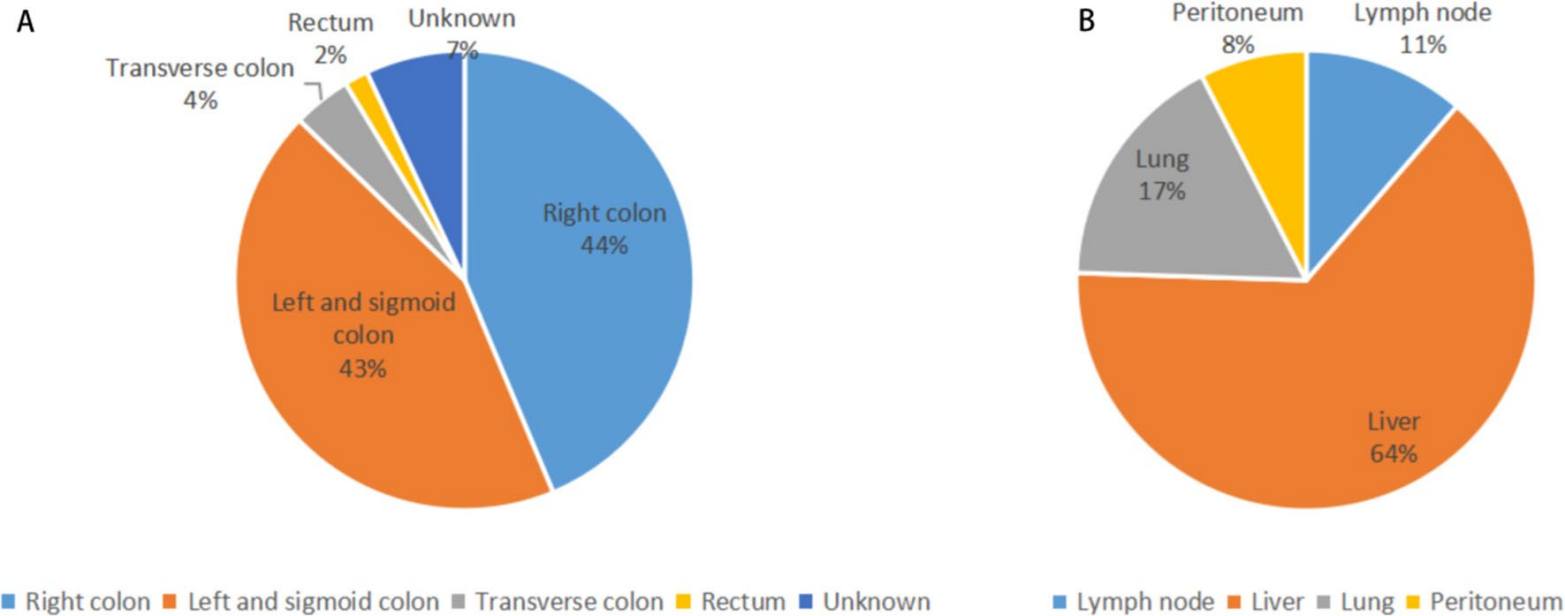
Percentage of tumor locations (**A**) and metastatic sites (**B**)

**Fig. 3:**
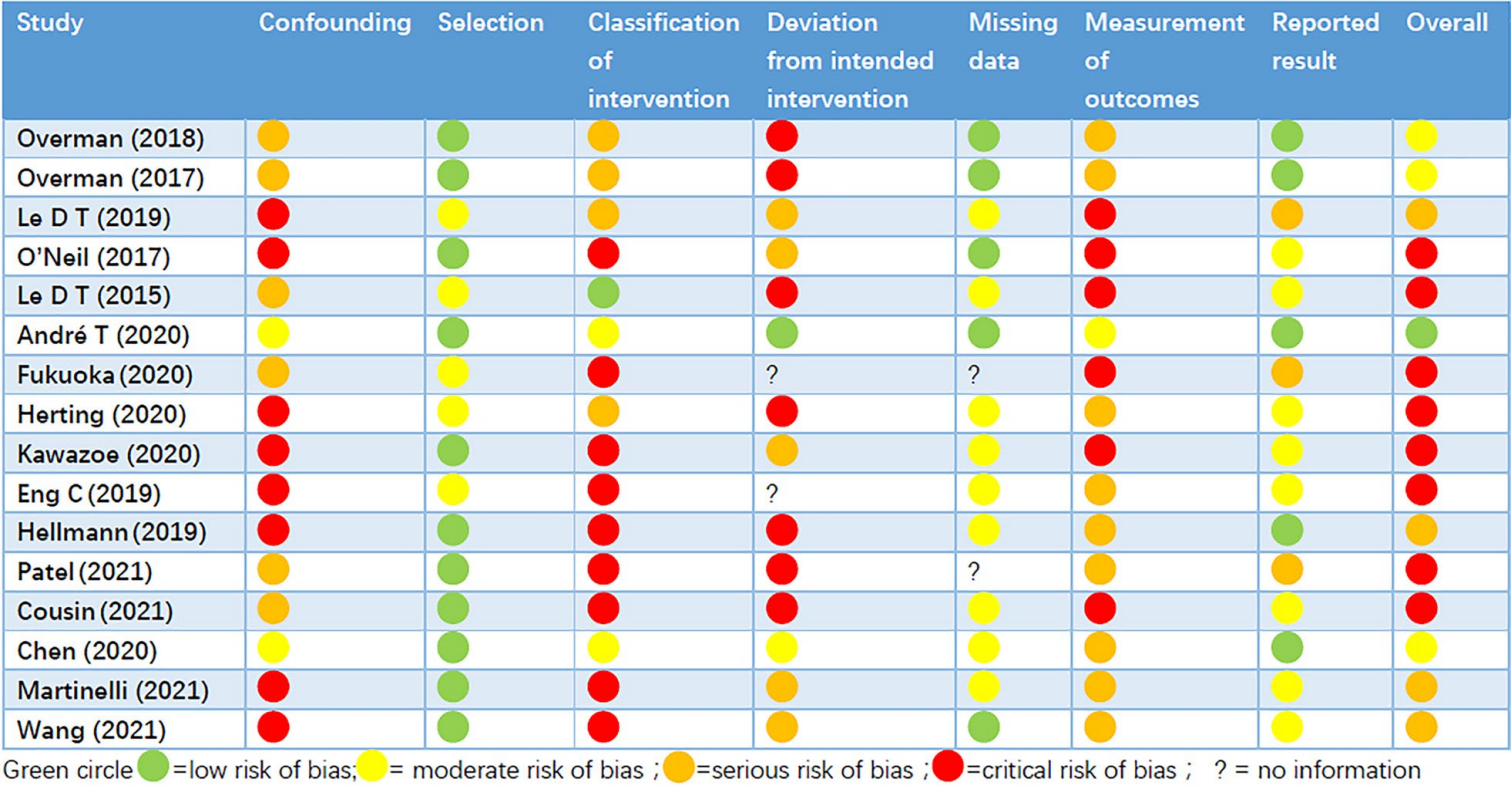
Assessment of risk of bias by area and overall

Sixteen studies including 1,503 patients were finally identified for inclusion into the study via full-text review and data extraction. The details of the selection process were in line with the PRISMA flowchart (Fig. 1). The characteristics of the 16 included studies are summarized in Table 1. Pie charts were drawn based on the primary tumor site and metastatic sites (Fig. 2A and B). The quality of observational studies was evaluated using the ROBINS-I tool. The detailed information of each study is shown in Fig. 3. The RCT studies of André T using the ROBINS-2 tool was low risk of bias and the study by Chen was moderate risk of bias.

**Table1:** Main characteristics of included studies

Author(year)	Case	Age	Genotype (N)				Microsatellite instability status	PD-L1 expression			Intervention methods
			WT	BRAF MU	KRAS MU	Unknown		≥ 1%	< 1%	Unknown	
Overman (2018)	119	58(21–88)	31	29	44	15	MSI–H 119	26	65	28	Nivolumab (PD-1) + Ipilimumab (CTLA-4)
Overman (2017)	74	53(44–66)	29	12	26	7	MSI–H 74	21	47	6	Nivolumab (PD-1)
Le D T (2019)	124	56(21–84)	17	14	46	–	MSI–H 124	–	–	–	Pembrolizumab (PD-1)
O’Neil (2017)	23	57(40–78)	–	–	–	–	MSI–H 1 MSS 22	23	–	–	Pembrolizumab (PD-1)
Le D T (2015)	32	54(24–79)	–	19	19	–	MSI–H 11 MSS 21	–	–	–	Pembrolizumab (PD-1)
André T (2020)	307	63(24–93)	69	77	74	90	MSI–H 307	–	–	–	Pembrolizumab (PD-1)
Fukuoka (2020)	25	55(31–77)	–	–	6	–	MSI–H 3 MSS 22	9	16	–	Nivolumab (PD-1) + Regorafenib
Herting (2020)	30	49(28–74)	21	0	14	26	MSI–H 2 MSS 9 Unknown 19	–	–	–	Pembrolizumab (PD-1) + Modified FOLFOX6
Kawazoe (2020)	50	58(25–79)	4	28	19	13	MSI–H 10 MSS 40	4	–	–	Pembrolizumab (PD-1) + Napabucasin"
Eng C (2019) A	183	58 (51–67)	258	9	99	–	MSI–H 3 MSS 170 Unknown 10	79	84	20	Atezolizumab(PD-L1) + Cobimetinib
Eng C (2019) B	90	56 (51–64)	128	3	49	–	MSI–H 3 MSS 83 Unknown 4	35	42	13	Atezolizumab(PD-L1)
Hellmann (2019)	84	56(23–79)	63	40	24	41	MSI–H 2 MSS 61 Unknown 21	–	–	–	Atezolizumab(PD-L1) + Cobimetinib
Patel (2021)	18	56(40–70)	–	1	12	–	MSS 18	–	–	–	Nivolumab(PD-1) + Rifluridine/Tipitaka (FTD/TPI)
Cousin (2021)	48	62 (26–83)	–	3	30	–	MSS 48	6	–	–	Aveluma (PD-L1) + Regorafenib
Chen (2020)	180	65 (36–87)	–	–	–	–	MSI–H 2 MSS 166 Unknown 12	–	–	–	Durvalumab(PD-L1) + Tremelimumab(CTLA-4)
Martinelli (2021)	77	–	48	19	19	3	MSI–H 3 MSS 71 Unknown 4	–	–	–	Avelumab(PD-L1) + cetuximab
Wang (2021)	39	52(37–69)	13	2	20	4	MSS 38 MSI–L 1	–	–	–	Toripalimab(PD-1) + regorafenib

*vs* Versus, *WT* wild type, *MU* mutant, *MSI-H* Microsatellite instability-high, *MSS* microsatellite-stable, *MSI-L* microsatellite instability-low

### Meta-analysis

#### Efficacy [15–30]

**Fig. 4:**
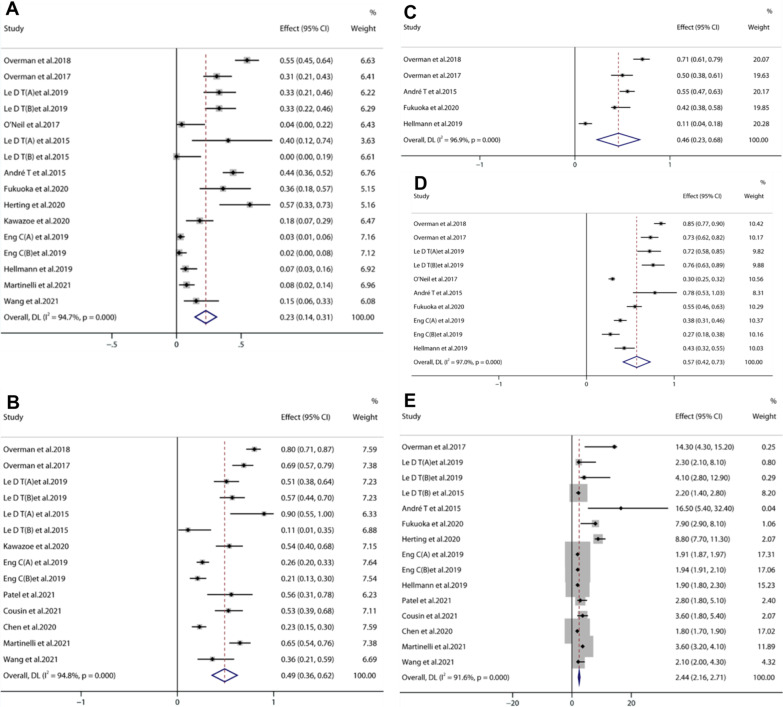
Forest plots showing the results of the objective response rate (**A**), disease control rate (**B**),1-year progression-free survival rate (**C**), 1-year overall survival rate (**D**), median progression-free survival (**E**)

The ORR of anti PD-1/PD-L1 therapy for advanced CRC was 23% [95%CI (0.14, 0.31) *P* < 0.001] (Fig. 4A). The DCR was 49% [95% CI (0.36, 0.62) P < 0.001] (Fig. 4B). The 1-year PFSR was 46% [95% CI (0.23, 0.68) *P* < 0.001] (Fig. 4C). The 1-year OSR was 57% [95% CI (0.42, 0.73) *P* < 0.001] (Fig. 4D). The median progression-free survival (mPFS) was 2.44 months [95% CI (2.16, 2.71), *P* < 0.001] (Fig. 4E).

#### Safety [15–30]

**Fig. 5:**
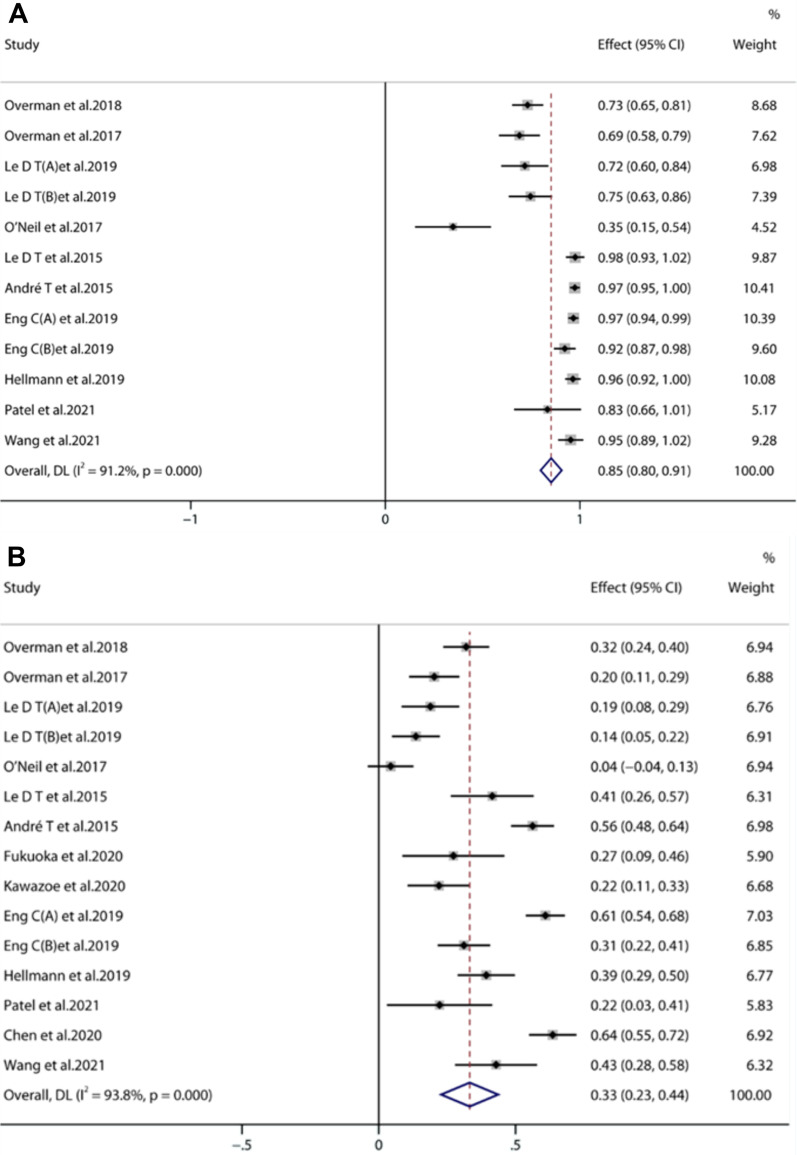
Forest plots of any grade TRAEs (**A**) and grades ≥ 3 TRAEs (**B**)

The incidence of any grade TRAEs associated with the treatment of advanced CRC with anti- PD-1/PD-L1 was 85% [95% CI (0.80, 0.91), *P* < 0.001] (Fig. 5A). The occurrence rate of grade 3 to 5 AEs was 33% [95% CI (0.25, 0.50), *P* < 0.001] (Fig. 5B). The most common AEs were diarrhea (36.0%), fatigue (32.82%), poor appetite (28.50%), nausea (25.10%), increased AST (22.46%), rash (22.37%), abdominal pain (20.60%), fever (19.88%), increased ALT (17.90%), hypothyroidism (12.62%), and pancreatitis (10.23%). Detailed descriptions of the adverse reactions are shown in Table 2.

**Table 2:** The incidence of grade1-2 TRAEs and grade ≥ 3 TRAEs

TRAE Name	Studies	Grade1-2	Grade ≥ 3
		Heterogeneity rate (95% CI) %	Heterogeneity rate (95% CI) %
Diarrhoea	15–21,23,24,26–28,30	Random 28 (18, 38)	Random 4 (2, 6)
Rash	15–20,21,23,24,27,28,30	Random 15 (9, 21)	Random 2 (1, 3)
Fatigue	15–20,21,23,24,26–28,30	Random 26 (17, 35)	Random 4 (2, 6)
Nausea	15–20,23,24,26–28	Random 21 (14, 28)	Random 1 (0, 2)
Poor appetite	18–20,23,24,26–28,30	Random 23 (14, 33)	Random 2 (0, 4)
Abdominal pain	15,17,20,24,26,28,30	Random 15 (8, 23)	Random 3 (1, 5)
Pyrexia	15–17,20,23,24,26,27,30	Random 16 (11, 21)	Random 2 (0, 3)
Increased AST	16,17,20,23,27,28	Random 16 (5, 27)	Random 4 (2, 6)
Increased ALT	15–17,20,27,28	Random 12 (5, 19)	Random 3 (2, 5)
Hypothyroidism	15–17,20,23,27,30	Random 11 (7, 16)	Random 1 (− 1, 2)
Pancreatitis	13,14,16,17,24,25	Random 7 (2, 12)	Random 5 (1, 9)

### Subgroup analysis

#### Microsatellite status [16–18, 23–25]

**Fig. 6:**
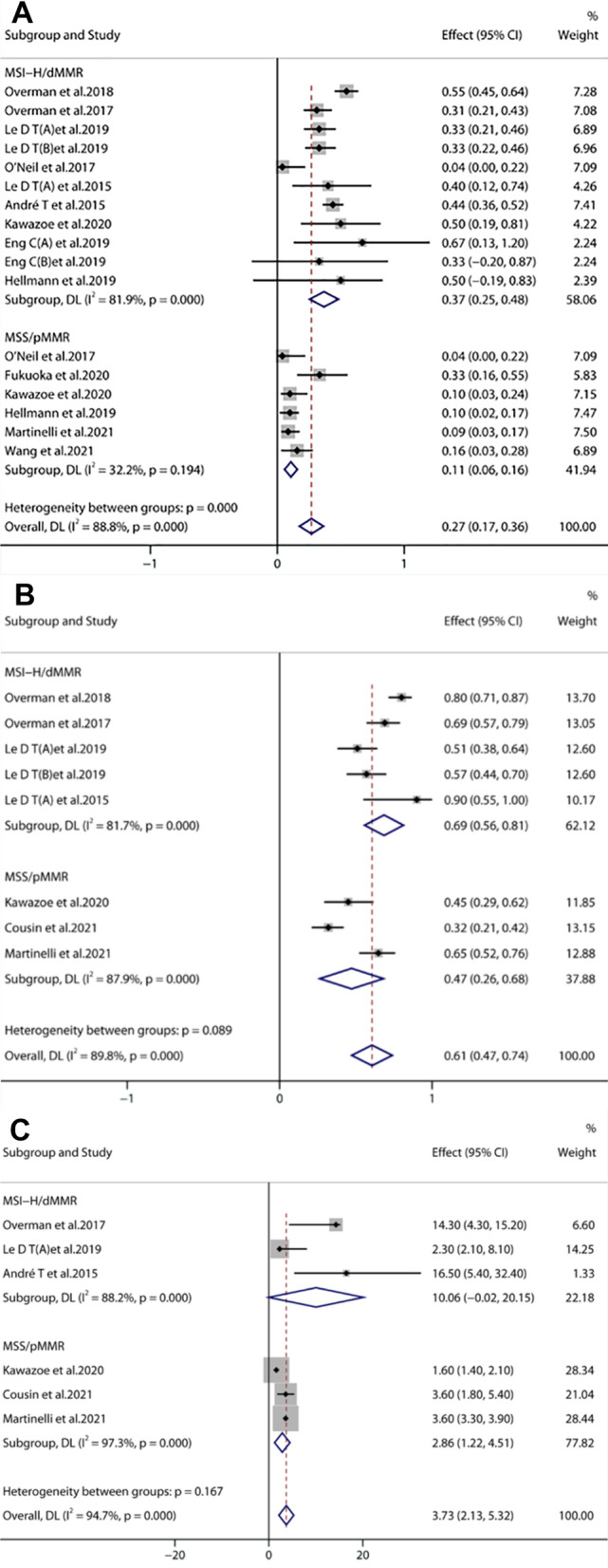
Forest plots of microsatellite status including ORR (**A**), DCR (**B**), and mPFS (**C**)

There were six studies analyzing microsatellite status. The ORR, DCR, and mPFS in MSI-H/dMMR patients were 37%, 69%, and 10.06 months, respectively, whereas those in microsatellite stable/mismatch repair proficient (MSS/pMMR) patients were 10%, 57%, and 2.86 months, respectively (Fig. 6A–C). The results indicated that anti-PD-1/PD-L1 therapy was associated with clinical benefit in more than one-third of MSI-H/dMMR mCRC patients. The ORR of MSS patients who did not respond to previous single-drug treatment reached 10% after immunotherapy combined with other therapies.

#### Different genotypes [16–18, 24, 25, 29, 30]

**Fig. 7:**
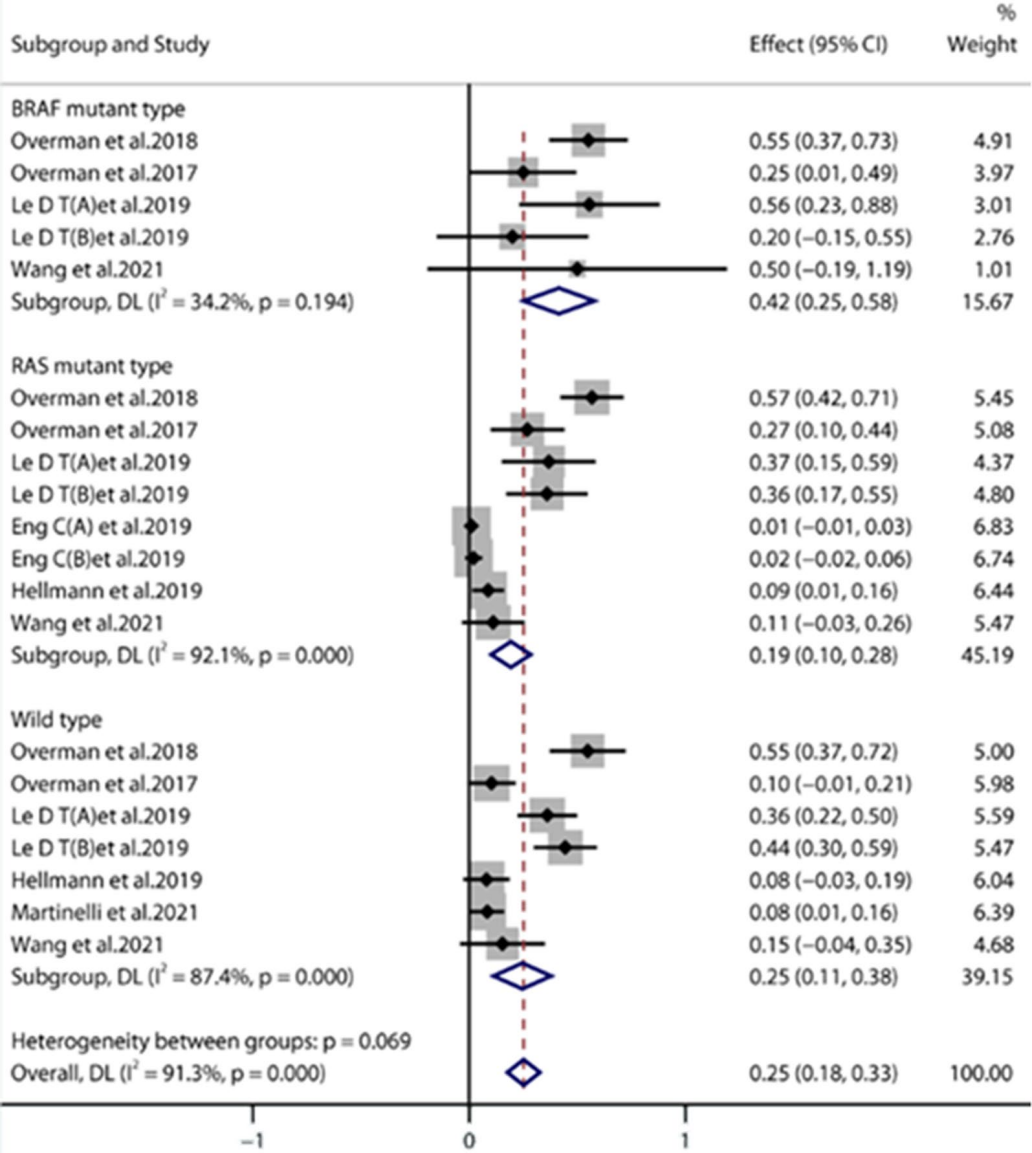
Forest plots of different genotypes of the ORR

Seven studies evaluated the ORR of patients with different genotypes. The ORR of the BRAF mutant subgroup, RAS mutant subgroup, and wild-type subgroup was 42%, 19%, and 25%, respectively (Fig. 7). The results indicated that immunotherapy was effective for BRAF mutant and KRAS/NRAS(RAS) mutant CRC, which may become a biomarker for the assessment of immunotherapy efficacy.

#### PD-L1 status [16, 18, 19, 21, 23, 24]

**Fig. 8:**
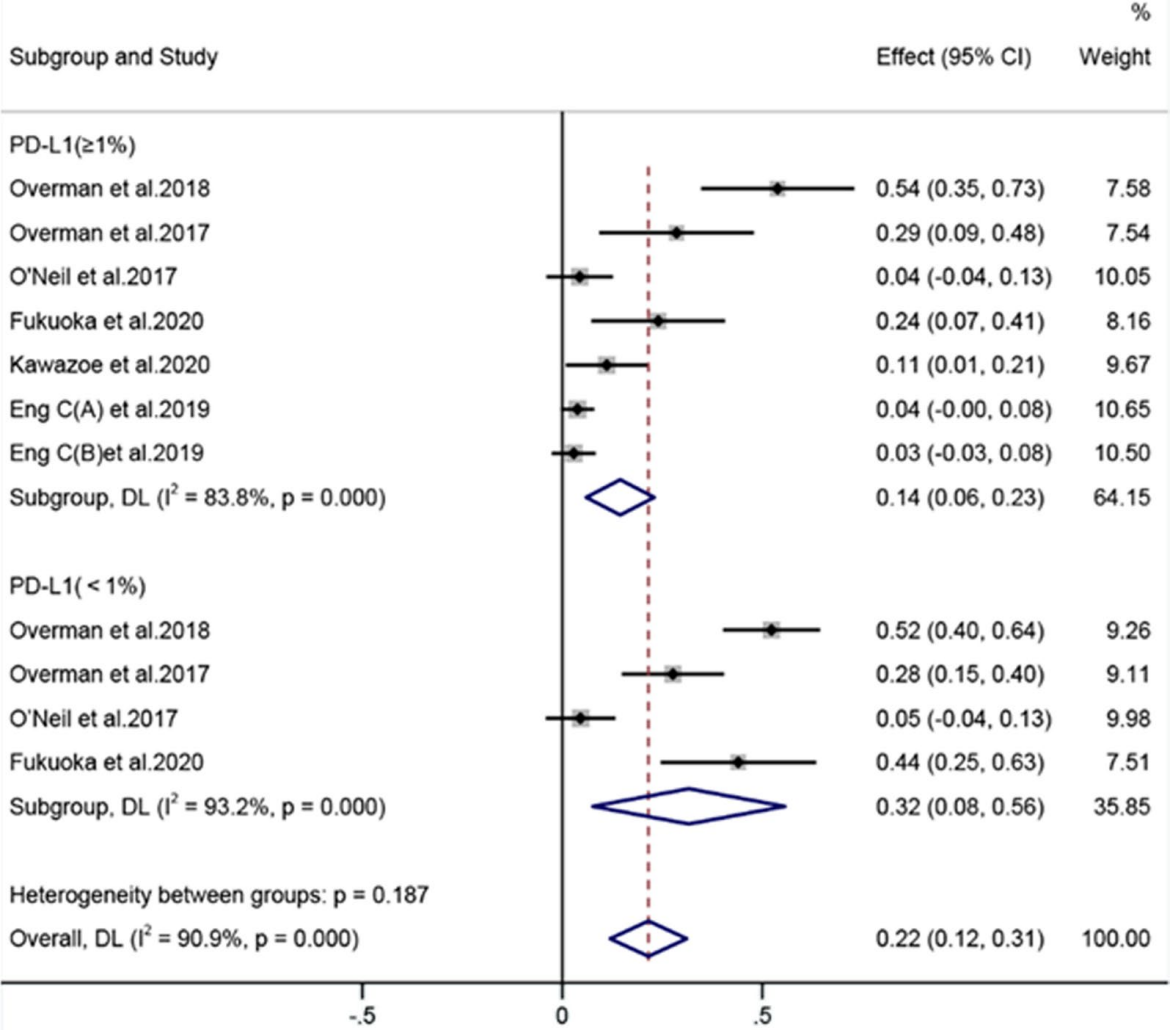
Forest plots of PD-L1 status of the ORR

Six articles analyzed the effect of PD-L1 expression. In patients receiving anti-PD-1/PD-L1 therapy, the ORR of PD-L1(≥ 1%) patients was 14%, whereas that of the PD-L1(< 1%) subgroup was 32% (Fig. 8). PD-L1 expression was positively associated with decreased ORR.

#### Publication bias and influence analysis

**Fig. 9:**
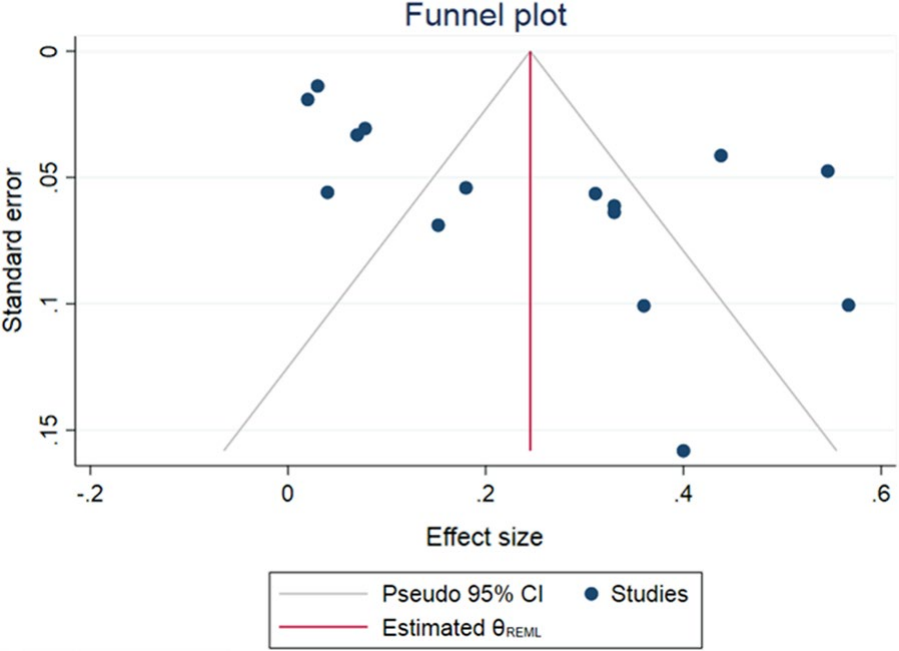
Publication bias of the overall ORR

**Fig. 10:**
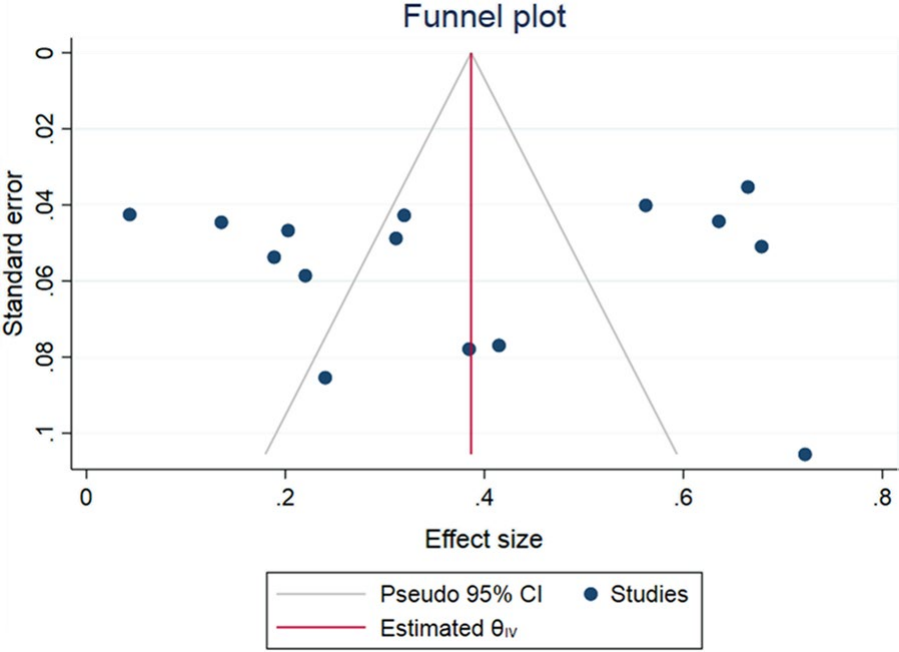
Publication bias of any grade adverse reaction

Publication bias Funnel plots were used to investigate the potential publication bias of the studies included in the meta-analysis. As shown in Figs. 9 and 10, the funnel plots were asymmetric, suggesting a medium risk of publication bias because of insufficient RCT articles. In the MSI-H/dMMR subgroup, we performed an influence analysis. The results indicated that the data of O'Neil were the source of high heterogeneity in our meta-analysis, and when this study was removed, the heterogeneity of the remaining 10 studies decreased from 81.9 to 45.1% (Additional file 1). In the MSS subgroup, however, influence analysis showed that the study by Fukuoka had an effect on heterogeneity, so significantly reduced heterogeneity was found when ignoring the Fukuoka study (Additional file 2).

## Discussion

The KEYNOTE016 study of pembrolizumab monoclonal antibody therapy showed that the ORR of the MSI-H/dMMR CRC, MSI-H/dMMR nonCRC, and MSI-H/pMMR CRC subgroups was 40%, 71%, and 0%, respectively. This suggested that patients with advanced dMMR CRC benefited from PD-1 inhibitor therapy, whereas patients with pMMR CRC did not benefit from anti-PD-1 therapy [31]. The 2018 ESMO meeting first reported that the ORR of MSI-H/dMMR CRC patients receiving first-line immunotherapy was 60% in the Checkmate 142 study. [16, 17], which indicated the probability of immunotherapy transition from third-line therapy to first-line therapy. This study was immediately followed by the KEYNOTE-177 study, in which patients were randomized to a pembrolizumab arm and standard first-line treatment group. This study further suggested that pembrolizumab improved survival significantly compared with standard chemotherapy [20].

In the present meta-analysis, the ORR of anti-PD-1/PD-L1 antibody therapy in the treatment of advanced CRC reached 23%. Subgroup analysis showed that the ORR of anti-PD-1/PD-L1 antibody therapy in the MSI-H/dMMR subgroup was 36%, whereas the ORR in the MSS/pMMR subgroup was 10%. This indicated that immunotherapy was emerging as the next frontier in the treatment of MSI-H/dMMR mCRC. Alternatively, combination treatment with immunotherapy agents and other methods is also promising for MSS/pMMR mCRC patients. Anti-angiogenic agents have shown a positive synergistic effect with immunotherapy by improving the tumor immune microenvironment, improving drug delivery, and facilitating immune cell responses [32]. The Japanese REGONIVO study showed an ORR of 33% in advanced CRC patients with MSS status who received nivolumab and regorafenib [21]. However, the North American REGNIVO Phase II study failed to replicate the Japanese results with an ORR of 7%. Because of its likely synergistic effect on the immune system, anti-EGFR/anti-PD1 combination treatment could be a promising therapeutic option for MSS mCRC patients. The AVETUXIRI study divided MSS mCRC patients into two groups receiving avelumab in combination with cetuximab. The results showed that the OS of the RAS WT and RAS MUT groups was 12.7 and 14.0 months, respectively [33]. Studies exploring the efficacy of immunotherapy in combination with MEK inhibitors or chemotherapy did not find a clear advantage in efficacy and safety compared with chemotherapy combined with targeted drugs in MSS tumors [22, 24]. The efficacy and safety of combination therapy require further exploration.

Identifying predictive biomarkers of the response to immunotherapy in mCRC is important. The CAVE study reported that cetuximab combined with avelumab achieved certain efficacy in wild-type RAS mCRC patients [34]. The present meta-analysis showed that the ORR of the BRAF mutant, RAS mutant, and wild-type subgroups was 42%, 19%, and 25%, respectively. Further studies are necessary to determine whether genotype status could be a predictive marker of a positive response. In addition, there is an ongoing debate about the prognostic role of PD-L1, with both favorable and unfavorable outcomes reported in various malignancies [35]. A meta-analysis by Cao et al. [36] showed that PD-L1 overexpression was associated with poor prognosis in patients with CRC. In this meta-analysis, the definition of positive expression of PD-L1 was PD-L1 expression ≥ 1% or a combined positive score (CPS) of ≥ 1. This meta-analysis showed that the ORR of immunotherapy was 14% in the PD-L1( +) and 32% in the PD-L1(-) subgroup.PD-L1 expression in CRC cells may be a predictive biomarker of response to immunotherapy. Previous research reported that PD-L1 was expressed in neoplastic cells (NCs) and tumor-infiltrating immune cells [37] and was associated with dMMR advanced CRC [38]. Therefore, it is important to clearly define PD-L1 expression levels according to tumor-infiltrating immune cells and tumor mismatch repair status.

Several biomarkers have been proposed and are currently being investigated. The Scoop study reported that patients with right-sided primary tumors had a higher ORR to anti-PD-1 treatment than those with left-sided primary tumors [23]. The right-sided colon was associated with a higher frequency of consensus molecular subtypes (CMS1) and greater infiltration of immune cells with high cytotoxicity than the left-sided colon [39], which might be one of the reasons for the different response to immunotherapy. The North American REGNIVO and China REGOTORI studies revealed that liver metastasis showed a poorer response to anti-PD-1 monotherapy than other metastases. The REGOTORI study also found that the intestinal flora may affect the efficacy of immunotherapy in MSS CRC [29]. In addition, tumor mutational burden (TMB) may be a biomarker for the response to immunotherapy. The CCTG CO.26 study showed that TMB is related to the efficacy of dual immunotherapy, and patients with TMB > 28 mts/Mb may benefit from immunotherapy [28]. These studies indicated that the discovery of novel biomarkers may widen the application of immunotherapy and bring new hope to CRC patients in the near future.

However, ICIs can cause a range of TRAEs affecting a multitude of organs, including skin, gastrointestinal tract, endocrine system, heart, lung, kidneys and the nervous system, and manageable safeties of anti-PD-1/PD-L1 were reported in various solid tumors, including melanoma, lung cancer, head and neck cancer, breast cancer, and urothelial carcinoma [40, 41]. Grades 3−5 TRAEs were observed in 33% of patients and 85% of patients had any grade of TRAEs. The most common AEs were diarrhea, fatigue, poor appetite, nausea, increased AST, rash, abdominal pain, fever, increased ALT, hypothyroidism and pancreatitis. This finding is roughly consistent with those of previous studies. However, it is worth noting that combination treatments might cause more TRAEs. Eng reported two of 179 patients treated with atezolizumab plus cobimetinib died of sepsis related to immune combination targeted therapy and one patient died of sepsis in the study of Hellmann, using atezolizumab combined with cobimetinib in solid tumors.

This meta-analysis had several limitations. First, the 14 studies included were single-arm studies. Although there were two RCTs, only the outcome of ICIs-related groups were included in our meta-analysis because of different interventions. Therefore, the results could be affected. Second, although subgroup analyses were performed, the heterogeneity was not significantly decreased. Therefore, the results of this study should be interpreted with caution, and further large randomized clinical trials are needed for verification.

## Conclusion

Despite the limitations of the included studies, the results of this meta-analysis indicated that immunotherapy has become an effective first-line treatment for patients with MSI-H/dMMR mCRC, and the combination of anti-PD-1/PD-L1 therapy with other therapies could be a useful strategy for the treatment of MSS/pMMR patients. The efficacy of immunotherapy was relatively low, underscoring the need to identify markers such as PD-L1 expression and different genotypes to predict the response to immunotherapy. High quality prospective studies and randomized controlled trials are needed to confirm these viewpoints.

## Supplementary Information

**Additional file 1**. Forest plot of sensitivity analysis in MSI-H subgroup.

**Additional file 2**. Forest plot of sensitivity analysis in MSS subgoup.

## Data Availability

Materials described in the manuscript, including all relevant raw data, will be freely available to any researcher wishing to use them for non-commercial purposes. Please contact Dr. Zhu, the corresponding author, for any inquiries for the data.
