# Supplementary figures and images for: Efficacy and safety of anti-PD-1/PD-L1 therapy in the treatment of advanced colorectal cancer: a meta-analysis
Source: BMC Gastroenterol. 2022 Oct 10;22:431. doi: 10.1186/s12876-022-02511-7 (PMC9549670; doi:10.1186/s12876-022-02511-7)

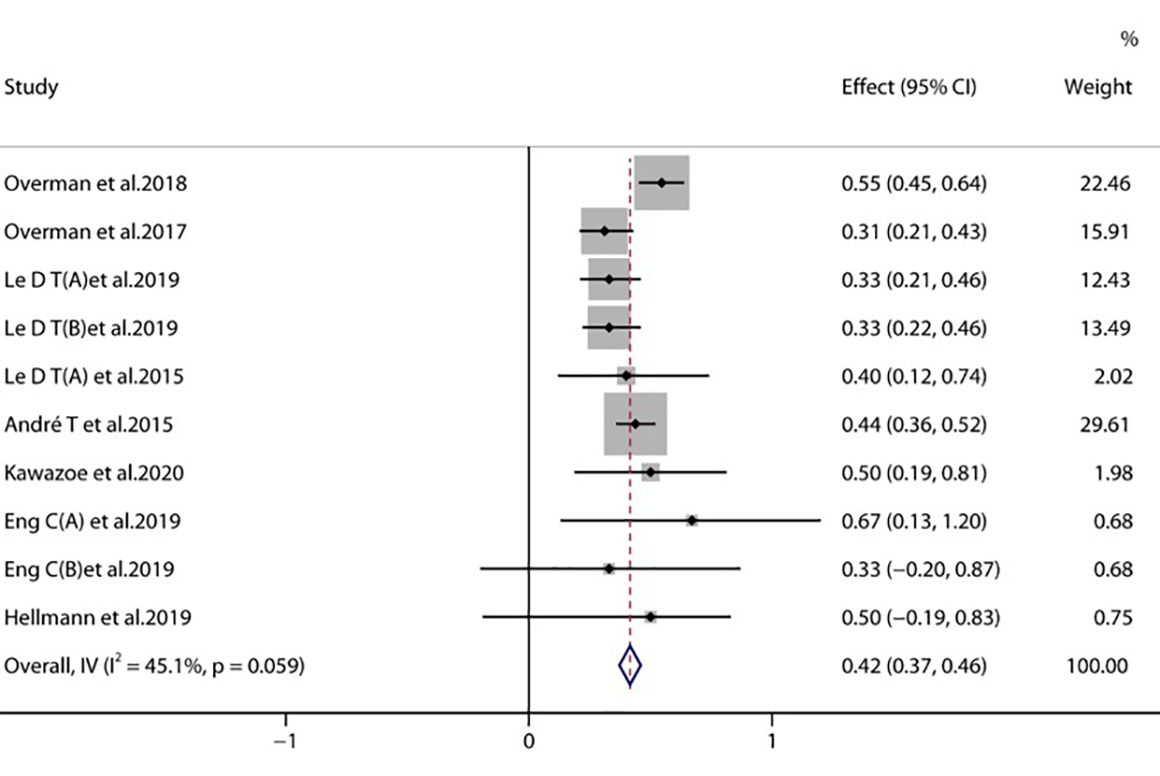

Supplement: Supplementary file 1 — Additional file 1. Forest plot of sensitivity analysis in MSI-H subgroup. [file 12876_2022_2511_MOESM1_ESM.tif]

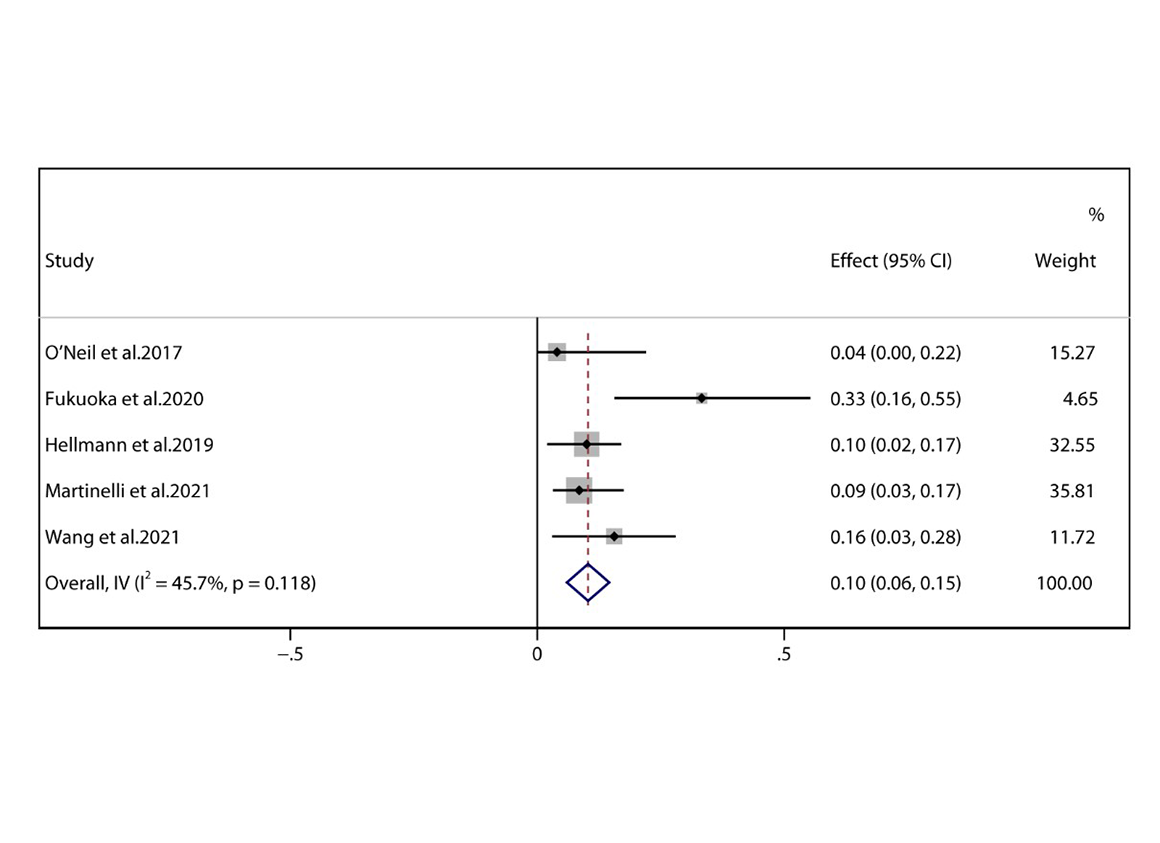

Supplement: Supplementary file 2 — Additional file 2. Forest plot of sensitivity analysis in MSS subgoup. [file 12876_2022_2511_MOESM2_ESM.tif]
